# Supplementary material for: The causal relationship between the human gut microbiota and pyogenic arthritis: a Mendelian randomization study
Source: Front Cell Infect Microbiol. 2024 Nov 26;14:1452480. doi: 10.3389/fcimb.2024.1452480 (PMC11629706; doi:10.3389/fcimb.2024.1452480)
Supplement: Supplementary file 1 [file DataSheet1.zip › mendelian test/ebi-a-GCST90027750.csv_leave_one_out.pdf]

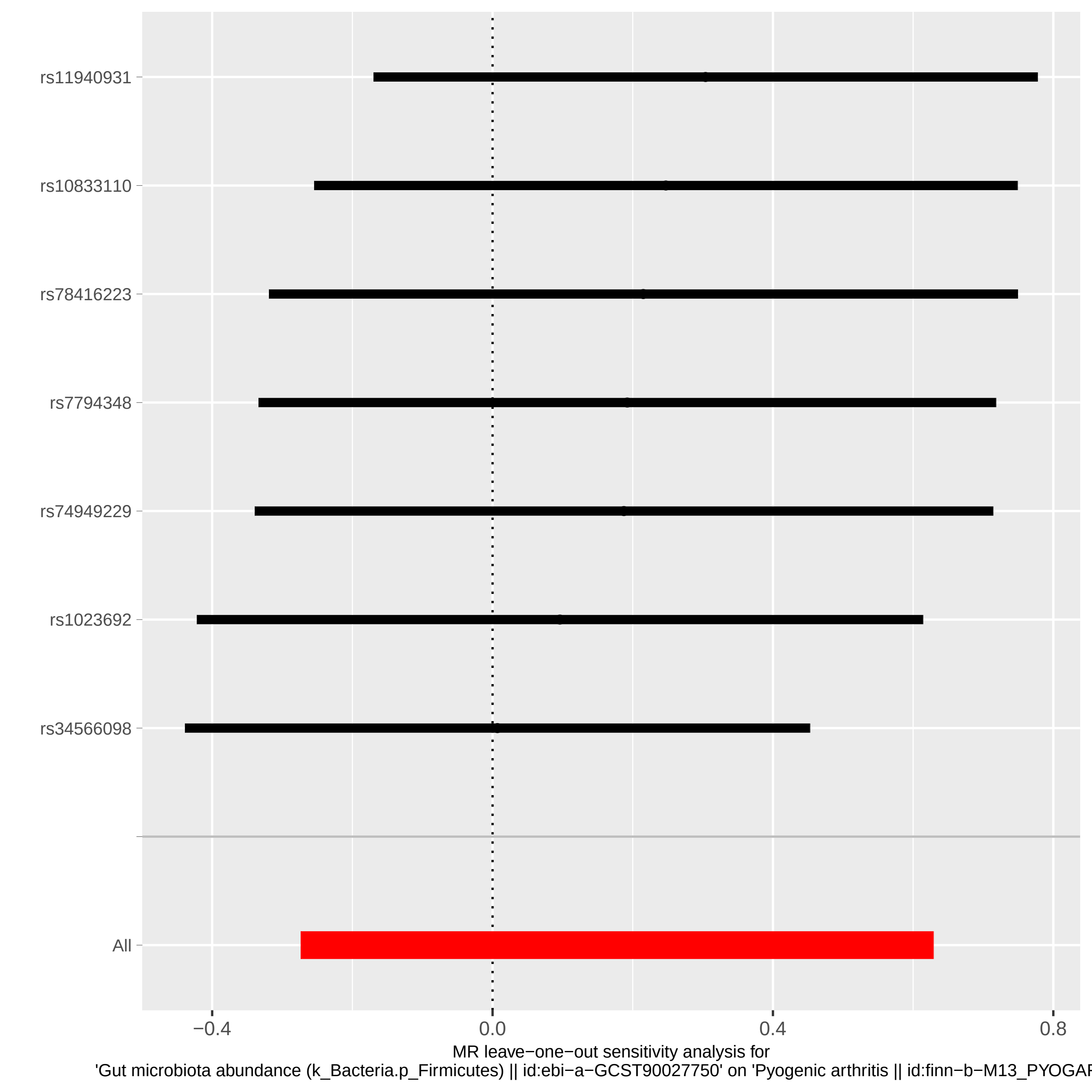

rs11940931
rs10833110
rs78416223
rs7794348
rs74949229
rs1023692
rs34566098
All
−0.4
0.0
0.4
0.8
MR leave−one−out sensitivity analysis for
'Gut microbiota abundance (k_Bacteria.p_Firmicutes) || id:ebi−a−GCST90027750' on 'Pyogenic arthritis || id:finn−b−M13_PYOGAR
